# Children’s needs during disclosures of abuse

**DOI:** 10.1007/s43545-022-00397-6

**Published:** 2022-06-28

**Authors:** Tara R. Ettinger

**Affiliations:** Department of Social Work, University of British Columbia, Kelowna, Canada

**Keywords:** Children, Youth, Child abuse, Disclosures, Child abuse investigations

## Abstract

A Narrative Literature Review was conducted providing a comprehensive overview of children’s barriers to disclose during investigations of child abuse. Patterns in the literature were categorized as themes and include: rapport and relationship with the interviewer, feeling in control and prepared, communication, physical abilities, mental health, environment, family dynamics, culture and individual uniqueness. Using a combination of a critical analyses approach and drawing from personal background experiences and knowledge in working with children during disclosures, the themes are expanded upon as a discussion that explores what children may therapeutically need during their disclosures of abuse within the forensic interview. Some practice implications are incorporated with the intent to generate further thinking about addressing children’s needs during investigations of child abuse. Working with multidisciplinary teams in Child and Youth Advocacy Centres is discussed and may be a resource for understanding children’s needs during disclosures of abuse.

## Introduction

Child abuse, also known as maltreatment, is common (World Health Organization 2020a, b). Child maltreatment is a global problem with serious life-long consequences and devastating impacts on all its victims, their families and society as a whole. In addition to the physical, social and psychological consequences of child maltreatment, the economical impacts from “hospitalization, mental health treatment, child welfare, and longer-term health [issues]” are tremendous (World Health Organization 2020a, b, p. 3). Most countries have policies responding to suspected cases of child abuse (ISPCAN 2008); these most often include law enforcement investigating the case and conducting child forensic interviews to help determine what occurred (Newlin et al. 2015). A child’s disclosure of abuse during the forensic interview can help substantiate the case, as well as heighten the ability for professionals to apply protective and healing interventions. However, literature demonstrates several factors that create barriers for children to disclose their experience(s) of abuse to professionals. Therefore, it is crucial for professionals involved in responding to child abuse to understand what children need and how to address their needs during their disclosures. The aim of this paper is to understand children’s therapeutic needs during disclosures of physical and sexual abuse within forensic interviews; specifically for investigations in Canada. However, the insight this paper offers into children’s needs during disclosures of abuse may be useful for professionals who work on cases of child maltreatment and are outside of Canada as well. The terms *abuse* and *maltreatment* are used interchangeably. The term *child(ren)* is used, rather than using both the terms child(ren) and youth, simply for the ease of reading. Respectfully, child(ren) refers to anyone under the age of 19. Multidisciplinary teams (MDTs) and Child and Youth Advocacy Centres (CYAC, sometimes referred to as Child Advocacy Centre, CAC) are discussed throughout this paper.

### Definition and prevalence

Child physical and sexual abuse are common worldwide (World Health Organization 2020a, b). Across all countries, the most common acts of child abuse involve physical abuse by parents or caregivers and sexual abuse defined as “incest, sexual touching or pornography” (International Society for Prevention of Child Abuse and Neglect [ISPCAN] 2008, p. 17). It is estimated that globally, nearly one quarter of children experience physical abuse and close to 20% of girls and 8% of boys experience sexual abuse (World Health Organization 2017). Most cases of child maltreatment occur when children are in a position of dependence on the person who is abusing them, which often makes maltreatment a hidden issue. These factors make it difficult to obtain a complete picture for the prevalence of child abuse in Canada (Department of Justice Canada 2002). Reported cases indicate that just over 30% of all Canadians aged 15 and older have experienced maltreatment during childhood, and that at least 40% of Indigenous people experienced childhood physical and/or sexual abuse (Afifi et al. 2014).

### The impacts of child abuse

The emotional effects of child abuse stretch far beyond the victim’s family, friends and communities, and its financial consequences are massive—in Canada, the cost is billions of dollars each year (deBoer, Rothwell, and Lee 2013; Statistics Canada 2017). Child maltreatment can deliver a life sentence of destruction for its victims (Public Health Agency of Canada 2008); children’s physical, emotional and social development become jeopardized as short- and long-term consequences (World Health Organization 2017). Studies such as the Adverse Childhood Experiences Study (ACE) have demonstrated an association between such childhood experiences and significant health and social problems across the lifespan (Felitti et al. 2019). The severe stress caused by child maltreatment is associated with a disruption in early brain development, and this can greatly impair further on-going development (World Health Organization 2020a, b). Consequently, maltreated children are at an increased risk for various behavioural, physical and mental health issues such as, depression, anxiety, post-traumatic stress, adolescent delinquency, obesity, drug and alcohol use, perpetrating or being a victim of violence, and other clinically significant mental health problems (Wolfe et al. 2001; World Health Organization 2020a, b). These behavioural and mental health consequences can then lead to further health detriments such as “heart disease, cancer, suicide and sexually transmitted infections” (World Health Organization 2020a, b, p. 3). Experiencing violence in childhood increases the likelihood of not graduating from school by 13% (World Health Organization 2020a, b). Children who suffer from child abuse are 41.2 times more likely to have attachment problems, three and a half times more likely to have poor emotional functioning and almost two and a half times more likely to face overall low life satisfaction (World Health Organization 2017).There is a clear link between children who have experienced child abuse and homelessness. Over the course of a year, the number of young people in Canada who become homeless and live in poverty is at least 40,000 (Homeless Hub 2021). In Canada, the majority of youth who are homeless reported that they experienced childhood trauma and abuse; therefore, most chose to leave an unsafe, abusive or neglectful situation (Homeless Hub 2017). Many experienced mental health concerns where their homeless situation was amplified by “slipping through the cracks” in the child protection, health and mental health systems (p. 4). Clearly, the trauma of child abuse is significant and can lead to negative consequences. Suspected cases require a thorough response so that protective and supportive measures can be implemented as early as possible and mitigate the possibilities for these unsettling outcomes.

### Investigating child abuse

In May 2016, World Health Organization endorsed a first ever global plan of action which committed to supporting countries and partners to implement evidence-based prevention and response strategies to cases of child maltreatment (World Health Organization 2021). This plan identified several strategies that have shown success in reducing violence against children such as, the implementation and enforcement of laws and strengthening responses and support services (World Health Organization 2021). Most policies worldwide will include criminal penalties for abusing a child and the provisions for removing a child in order to protect him/her from further abuse (ISPCAN 2008). In Canada, child abuse is identified as a health issue with child welfare and criminal justice implications (Government of Canada 2017). Every province requires mandatory reporting of all forms of known or suspected child abuse. For instance, under British Columbia’s *Child, Family and Community Service Act* (1996/2017), anyone who has received a disclosure of abuse from anyone under the age of 19, or has reason to believe that a child has been or is likely to be abused, must report the abuse.

*The Criminal Code of Canada* (1985/2017) requires law enforcement investigations for all cases of suspected child abuse. Investigation can determine whether a child has been harmed; and if so, to justify protection and medical strategies, discover what short- and long-term support the child may need, identify a potential offender and collect evidence for court. Conducting a forensic interview with the child is most commonly at the beginning of an investigation and can be the most significant intervention for gaining accurate information when abuse is suspected (Newlin et al. 2015). Further assessment can also occur within the forensic interview to better understand the safety of the child’s living situation and identify the need for medical treatment and/or psychological care (Government of Canada 2018a, b, c, d). Even though there is no one standardized practice, forensic interviewers need to be well trained (Newlin et al. 2015). Throughout the years of research and practice, greater insight in how to elicit quality evidence from child witnesses during forensic interviewing has been gained (Powell 2013; Price and Roberts 2011). The development of trauma-informed practices has helped professionals who work with individuals who have experienced trauma, as with those who have experienced child abuse (Urquhart, Jasiura, TIP Project Team, and TIP Advisory Committee 2013). Increased knowledge regarding abuse trauma and system-induced trauma has resulted in the development of better responses in child abuse investigations such as those in CYACs that conduct investigations in a child-friendly building and use a multidisciplinary and wraparound service approach (Department of Justice Canada, 2013; Shaffer et al. 2018). The MDTs in child abuse investigations began in the 1980s to increase communication and information sharing among investigative agencies (Department of Justice Canada 2013; Young and Nelson-Gardell 2018). More studies are understanding factors that increase the prevalence for sexual abuse disclosures in forensic interviews such as the child’s age and gender (Azzopardi et al. 2019; Lippert et al. 2009).

#### Informal disclosures

Children who have been abused often have difficulties telling this to another person. Particularly in cases of sexual abuse; many children never do share this information (Townsend 2016). When a child does disclose, it is important to differentiate the context in how it occurred because the situation for the child will be very different (Anderson 2016). McElvaney et al. (2012) noted Jones’ (2000) language for identifying two different types of disclosures. The first type is the informal disclosure where the child tells about the abuse to someone in their life. McElvaney et al. (2012) discussed controversial studies regarding who the most common receivers of informal disclosures are. They identified that recipients are generally those that the young person trusts and is close to such as, a parent, friend, sibling or a counsellor. There are many reasons why a person who received an informal disclosure of abuse may not report it. These circumstances mostly relate to people’s own situation, feelings, beliefs or their level of knowledge about child abuse (Government of Canada 2019).

Literature continually demonstrates how informal disclosures can be frequently delayed. It appears that making a disclosure often resembles an emotional and developmental process that involves both an unconscious and conscious contemplative undertaking where children may weigh the pros and cons in revealing their truth (Alaggia et al. 2019; McElvaney, 2008; McElvaney, Green, Hogan 2012). For many children and youth, “the benefits of telling need to outweigh the costs of telling” (Brennan and McElvaney 2020, p. 107). There are many factors why a delay in disclosure or even never telling about the abuse, are common. Issues such as shame, self-blame and fear are common factors that are continual barriers for children to informally disclose abuse (Alaggia et al. 2019; Allnock and Miller 2013). There is a lot of literature that repeatedly tells us that children need help in revealing the abuse that happened to them (Brennan and McElvaney 2020; Goodman-Brown et al. 2003; McElvaney et al. 2012). Some suggestions were for more effort to be placed on public awareness, education and other ways in which society can encourage children to talk about child abuse (Leclerc and Wortley 2015; Paine and Hansen 2002). Brennan and McElvaney (2020) concur that all children need to have educational opportunities about sexual behaviours to help them understand “what is okay and what is not okay” (p. 111). However, these authors placed emphasis on the emotional needs of children when making disclosures. They indicated how children psychologically need to tell, but they also need to have the right opportunity to tell. Children who have experienced abuse go through a process of first “recognizing the behaviour as abusive…[then experience] a build-up of psychological distress that overwhelms the child's own resources in coping with this distress” (Brennan and McElvaney 2020, pg. 101). McElvaney et al (2012) talked about a *pressure cooker effect* for why an informal disclosure may occur. Their analogy implies that children experience an inner turmoil and need to tell about the abuse; the overwhelming mix of emotions causes an immense build-up of stress. Brennan and McElvaney (2020) expressed how children need adults to respond to them appropriately and adequately by intervening, noticing and addressing their distress, and then asking them the right questions that will facilitate their disclosure.

There are several ways that an informal disclosure can occur. *Discovery* is when another individual recognizes the signs and symptoms of abuse (such as signs of injury or trauma) and reports it (Government of Canada 2018a, b, c, d). *Accidental* is when the child is not completely ready to tell but the disclosure 'slips out’ (Government of Canada 2018a, b, c, d). When the child actually tells someone about the abuse, this would be identified as o*n purpose* (Government of Canada 2018a, b, c, d), and these types of disclosures can occur during a forensic interview as well.

#### Formal disclosures

A formal disclosure is when a child tells about their abusive situation to a professional during a forensic interview. Young victims often tend to be the only eyewitness to the abuse and the most accurate information, most often, is a child’s formal disclosure given during the forensic interview (Alonzo-Proulx and Cyr 2016; Lyon et al. 2012). If a child does not formally disclose during the forensic interview, it can potentially limit the ability for professionals to protect or provide healing interventions and unfortunately, may result to longer or repeated abuse. A forensic interview can be quite intimidating for the child as it usually places him/her in an unfamiliar situation with an unknown professional (Anderson 2016). Some children identify how a disclosure during a forensic interview may have serious consequences for them (Anderson 2016). Azzopardi et al. (2019) examined the prevalence of sexual abuse disclosures in forensic interviews with children under 18 years for example. These authors concluded that more than one third of the children do not disclose during a forensic interview and that there are several issues that may have influenced the disclosure. Numerous factors can interfere with children’s effective disclosure of abuse during the forensic interview (Fontes and Plummer 2010). It is important to have an understanding of these factors. Such knowledge has not only allowed interviewing strategies to evolve, but it may further our insight to understanding children’s therapeutic needs during their disclosures of abuse.

**Methodology**

### Purpose

The purpose of the paper was to explore literature regarding children’s barriers to disclose during investigations of child abuse within the Canadian child welfare system in order to explore the question: *What are children’s therapeutic needs during disclosures of physical and sexual abuse within forensic interviews?*

### Objective

The objective was to conduct a Narrative Literature Review and provide a comprehensive overview of children’s barriers to disclose during investigations of child abuse to reveal what they need during interviews.

### Method

A review of the literature was conducted to identify children’s barriers to disclose during investigations of child abuse. The aim was to look for factors that would create challenges for children during disclosures of abuse within the forensic interview. The next step was to look for patterns regarding the challenges and categorize these *findings* into themes. Using a combination of a critical analyses approach and drawing from my background experiences and knowledge in working with children during disclosures, the themes were expanded upon into a *discussion* to explore what children may therapeutically need during their disclosures of abuse during the forensic interview. Some *practice implications* were incorporated with the intent to generate further thinking about addressing children’s needs during investigations of child abuse.

The initial search was performed through Google Scholar and British Columbia (UBC) Online Library. The process followed a method of investigating literature through English language, published, peer-reviewed reviews, empirical studies, books, research papers or government documents regarding child abuse and factors impacting disclosures during the forensic interview. Open sources relevant to the area of study were included in the review process. The majority of the literature was published between 2012 and 2022. However, some sources reviewed were published between 1998 and 2011 due to the current relevance that they demonstrated regarding children’s needs during disclosures of abuse. As well, it was interesting to note how some of the issues to disclose have been continuous issues throughout history. The search strategy involved utilizing various search strings such as: (child*) AND (youth) AND maltreatment/abuse AND (investigation*) AND (disclosure*) AND (barriers). Generally, if the title, abstract and keywords demonstrated the words in the search strings, the literature was reviewed. Some of these sources led to additional literature sources by exploring some of their citations/references; these were also reviewed for relevance. Literature was deemed relevant if it examined or discussed a variety of children’s experiences during the forensic interview that created challenges for them. This included literature that explained children’s feelings or factors that demonstrated to have an influence on their behaviour and ability to disclose. Such literature was believed to meet the objective of this paper. Literature that did not discuss issues of disclosures in cases of child physical and/or sexual abuse/maltreatment or demonstrated factors that would impact disclosures, the literature was extracted. Literature that was unable to be fully accessed by author could not be included.

## Findings from the literature

The combined literature demonstrated common influencing factors during children’s disclosures of abuse that are critical to understand. These factors need to be effectively addressed within the practice of forensic interviewing. Following are the nine factors:

### Rapport and relationship

Literature spoke to the importance of comfort between the interviewer and the child, in enabling them to talk about their abuse. O’Donohue and Fanetti (2016) expressed how the ability to quickly build effective rapport with children is the most crucial interviewer skill. They stated that when children feel nervous, frightened or intimidated, they will more likely “shut down” and “refuse to talk” (p. 185). Rapport is needed for the child to feel supported, empowered and encouraged in the relationship to speak up when they do not understand a question, or to say if they do not know the answer (Kebbell et al. 2006; Lyon et al. 2012). Allnock and Miller’s (2013) study demonstrated that the majority of youth interviewed regarding their cases of abuse identified a need for a better relationship with the interviewer; overall, they found disclosing their abuse to the police an uneasy and unpleasant process. The UK, National Police Chiefs Council conducted an informative survey that demonstrated how young people felt anxious and intimidated when they saw law enforcement and that they did not want any contact with them unless “it was through the right channel and with the right content” (Griggs 2017, p. 1). Even though the study focussed on children in the UK and seeing police in general, it provides great insight into how a child may be feeling going into a forensic interview.

Children need interviewers who demonstrate patience, calmness, sensitivity, warmth and genuiness to build a rapport (Themeli and Panagiotaki 2014). A study that examined forensic child interviewers in Sweden demonstrated how common strategies to build rapport involved showing interest and deep listening, talking with the child about her/his interests and to start building rapport in the waiting room to enhance the building of trust and comfort early and prior to the interview (Magnusson et al. 2020). Wood and Garven (2000) discussed a study in their review that suggested how a proficient rapport between interviewer and child may sometimes make the difference between disclosure and nondisclosure; their research showed how children tend to give more accurate statements when interviewed in a warm, supportive manner. Establishing a good rapport right from the beginning of the interview is crucial so that the child feels accepted, comfortable and suffers less anxiety. Even though such significance is placed on the need for a strong rapport with the child, many interviewers fail in it and try to move too quickly into the next phase of the investigation. (Themeli and Panagiotaki 2014).

### Staying in control and feeling prepared

Literature reflected the importance for the child’s need to understand the interview process and to feel a sense of control of the conversation in order to be able to talk about the abuse. During forensic interviews, children need to remain informed, feel they are the ones in charge of the conversation and go at a pace that works for them (Davies and Westcott 1999; Gilligan and Akhtar 2005). If children feel apprehensive about what is occurring, their ability to retrieve information from their memory will decrease (Kebbell et al. 2006). In Ungar et al.’s (2009) study of Canadian youth’s experiences of disclosing abuse, when interviewees felt the interviews were unmanageable, it would limit their ability to feel safe. This would, therefore, impact negatively on their ability to disclose the abuse. Alaggia’s (2010) study of disclosure experiences with child abuse survivors, identified that they wanted to have a sense of ownership of how others interpret their story or situation; going into the interview, they would often fear the possibility of being perceived a certain way. “Youth want control over any disclosure process” (Ungar et al. 2009, p. 705).

### Communication

Literature revealed the interviewer’s need to have a good awareness of the interviewees’ vocabulary skills and their understanding of abuse, in order to have a good investigative interview. Children’s ability to participate in strong investigative interviews decrease when interviewers use words or sentences that are above the children’s level of comprehension (Wood and Garven 2000). Even in cases with adolescents, who can often resemble adults and can be expected to have the verbal and cognitive abilities as such, may fail to feel comfortable in an interview due to miscommunication (Newlin et al. 2015). Korkman et al. (2008) indicated in their study how interviewers often felt frustrated or sad when they were unable to get interviewees to discuss their abuse; in most cases this was due to them not being able to use language or questioning that suited their ability. Sometimes children may not understand certain concepts that are relevant within forensic interviews, such as aspects of time or spacial relationships. In such situations, interviewers must be able to assess the child’s comprehension level or communication ability and then adapt their ways of asking questions (Stakić 2019).

It was suggested that an ideal time to assess the child’s linguistic abilities is during the rapport building stage, as it is before the real substantive portion of the interview (Rohrabaugh, London and Hall, 2016). Many authors pointed to the importance of integrating a preplanned investigation process, thus, allowing for a tailored interview to meet the communication needs of the child (Cederborg et al. 2008; Davies and Westcott 1999; Kebbell et al. 2006; Korkman et al. 2008; Schaeffer et al. 2011; Wood and Garven 2000). Stakić (2019) demonstrated the ability to create conditions for children in providing reliable narratives through strategies such as pre-interviews (before the forensic interview). This strategy is undertaken by a MDT to plan, determine goals and tasks and adjust the conditions of the interview to meet the needs of the child.

### Developmental abilities/limitations

Several authors addressed how different abilities and/or impairments could create obstacles to a collaborative interview between interviewer and child. For instance, different congenital factors can compromise children’s memory function (Peterson, Jones, Stephens, Gözenman and Berryhill, 2016). Consequently, children may have challenges retelling events during forensic interviews if they have certain medical, developmental or mental health conditions. Memory and cognition are also sensitive when exposed to alcohol and drugs, keeping in mind that exposure can occur prenatally (Peterson et al. 2016). Kebbell et al. (2006) highlighted how interviewing children who have disabilities is a complex process and few investigative interviewers know how to assist children who have intellectual and/or physical impairments. Interviews that involve children who have disabilities requires a process that “adapt[s] to the child’s communication style and limitations, and allow adequate time to gather information” (Newlin et al. 2015, p. 5). Information about the child’s presenting issues regarding disabilities, development and medical issues becomes a crucial part of being able to communicate effectively with a child during a forensic interview. Children with intellectual disabilities will need more time to: (a) understand the task at hand; (b) understand the question; (c) process the question; (d) retrieve information from memory and (d) put thoughts into words—or communicate in some other way, if they cannot find the word or have difficulty speaking (Kebbell et al. (2006). Investigative interviews with children who have limitations should involve such prior knowledge so that a tailored interview can be prepared to encompass physical or learning impairment needs (Davies and Westcott 1999). Cederborg et al.’s (2008) study involving forensic interviewing with children with intellectual disabilities, stressed the significance of being informed of children’s unique characteristics, abilities and limitations before interviewing them. They indicated how prior knowledge allows interviews to incorporate specific techniques and facilitate needs, possibly enhancing the ability to gain the disclosure. This strategy would allow additional insight into any special accommodations that may be needed to create the safer, more comfortable environment that will enhance the relationship between interviewer and interviewee (Newlin et al. 2015).

### Mental health

Abuse is a traumatic experience for children (Newlinet al. 2015). Investigators of child abuse need to take into account how the initial trauma resulting from the abuse impacts them. The stress from trauma may affect children’s memory development and communication encoding and retrieval skills that are needed to describe the abuse (Hritz et al. 2015; Middleton 2017). Children’s individual dispositions, temperaments and attachment styles have the ability to influence different degrees of stress that is placed on them as well (Hritz et al. 2015). Therefore, when children have been overly frightened, are still suffering from shock, or are still trying to process their traumatic experiences, they “may not be effective reporters in a forensic interview” (Newlin et al. 2015, p 6).

#### Trauma informed

This practice refers to incorporating the understanding of past and current experiences of trauma into all service delivery models (Poole 2013). The goal is to not re-traumatize, rather to promote health and healing. Langballe and Schultz (2017) recommended that victims who have suffered severe trauma should not even express thoughts and feelings during the first month after the event, as doing so may disrupt their essential and natural recovery process. Milam et al. (2017) expressed how trauma-informed should encompass a victim-centred practice as well. This approach highlights children’s need for a genuine rapport and feelings of trust with the professional. Milam et al. (2017) stated that the process should not aways be about getting the facts about the case; “not building rapport and trust creates the risk of re-traumatization and damage to the working relationship” (p. 40). Stackle et al. (2021) conducted a study to test their trauma-informed interviews against an already established interview technique. Even though the two techniques did not demonstrate any significantly different outcome, the trauma-informed approach did not take away from the efficacy of the standardized forensic interview protocol. This study may encourage further research regarding approaches that incorporate other techniques to better address children’s trauma, anxiety and stress during interviews.

There are additional mental health associations with children’s nondisclosures, such as feelings of shame, guilt, embarrassment, depression and suicidal thoughts (Allnock and Miller 2013). In Ungar et al.’s (2009) findings, youth anticipating the consequences that could follow disclosure intensified their fears. In their attempts to avoid overwhelming or frightening experiences, it is not uncommon for children to display behaviours such as distracting, dissociating, denying, or refusing to answer questions (Berson and Meisburger 1998). Alaggia’s (2010) study also addressed fears about disclosures—some feared being perceived differently or blaming themselves for the abuse. The young people in McElvaney’s (2008) research indicated they had emotional difficulty when they tried to disclose abuse; they felt afraid, implying that it was easier for them to keep it a secret. Understanding the underlying issues of the children’s mental health is paramount in planning the investigative interview and in being able to tailor it to meet their individual needs (Davies and Westcott 1999). Newlin et al. (2015) expressed being flexible for the timing the interview or to allow multidisciplinary intervention if a child’s mental health indicates that he or she is not capable of the interview yet. Barber Rioja and Rosenfeld (2018) explained a critical component of the forensic interview is being able to understand the mental status of the child and discussed an examination strategy to determine this. These authors explained how this method can involve a standardized-type of questioning or as an informal evaluation through observation during the interview. Regardless of method, the objective is to accurately describe the child’s behaviours, emotions and thoughts, particularly in comparison to the presence or absence of a mental disorder. Obviously, the forensic interviewer him/herself would need extensive training to understand and detect mental health disorders if there was no mental health clinician assisting during the interview. Involving mental health professionals as consultants within the forensic interview or as support alongside the criminal justice process itself, however, have been discussions for debate (Herbert et al. 2018). Barber Rioja and Rosenfeld (2018) pointed out that in the absence of an adequate cultural understanding, however, observations can result to misinterpretation, resulting in a faulty conclusion. Thus, demonstrating the importance to understand the influences of culture as well for children’s disclosures.

### Cultural awareness

Several authors addressed culture as a factor that could influence children’s willingness or ability to disclose abuse. Alaggia’s (2010) study demonstrated how children grow up learning certain expectations based on cultural messaging, which moulds their communication styles and can influence disclosures. Hritz et al. (2015) described this influence as a “cultural force” that can weigh on a child in their decision whether to disclose. Cultural groups that would have a higher prevalence of trauma in our society or suffer result of intergenerational trauma can negatively impact future generations in dealing with childhood trauma.

#### Culturally safe practice

A cultural-safe approach recognizes the impacts of our colonization histories and the essentialness for a trauma-informed practice. **“**Cultural safety [is] a preferred way of working as professionals [and the] need to decolonize our professional practice” (Thompson and Taylor 2021, p. 1). The first step in creating a culturally safe practice is having awareness. For our First Nations, Inuit and Métis Peoples in Canada, the trauma from colonization has left residual effects for Indigenous children and youth today, which can be seen in the high rates of mental health, addictions, violence and suicide (Bourassa et al. 2015; Sinhaa et al. 2013). Indigenous children and youth continue to be disproportionately overrepresentated within the Canadian welfare system and child maltreatment investigations (Blackstock et al. 2004; Government of Canada 2018a, b, c, d). Research suggests there are reoccurring patterns of child abuse across generations, however, understanding all the factors involved is an extensive and complex undertaking (Brokenleg 2012; Widom et al. 2015). Brokenleg (2012), a psychologist, member of the Rosesbud Sioux Tribe and an author in the area of trauma, resilience and Native American studies, reiterated how historical trauma can be cumulative and repeat across generations. He indicated how trauma has shaped society of Indigenous communities and the lack of healing for one generation can create problems for the next, having an effect on every Indigenous person. “It sculpts how we think, how we respond emotionally” (p. 10). Colonization results have created a multi-generation of grief, trauma, displacement and an understandable distrust for law enforcement and child protection workers (Blackstock and Trocmé 2005; Indigenous Services Canada 2018). Being trauma-informed and having knowledge regarding the maltreated child’s family history of trauma and/or intergenerational trauma is a significant factor during child maltreatment investigations (Pence 2011). A better understanding of current abuse disclosures from children who are from families that experience intergenerational trauma would be of benefit for investigational processes. More importantly, early intervention and response strategies that can disrupt harmful intergenerational outcomes. Professionals need to stay current about cultural awareness and to view each child’s cultural experience as unique, for there are many differing cultural contexts among children and adults (McElvaney 2008). Gilligan and Akhtar (2005) expressed the same view that professionals involved with child abuse cases must take cultural impacts into account and design their service to meet the needs of the child and allow flexibility in making disclosures. McElvaney (2008), however, discussed how agencies encourage little intervention prior to the formal investigative interview. She pointed out that response times from initial disclosure to formal disclosure vary considerably and raised the question of the professional and ethical difficulties in discouraging a child who needs to connect or to talk before the interview takes place. Some interview protocols recommend the interview be adapted according to the child’s cultural background and preferred language. Therefore, the interviewer should contemplate if an interpreter is needed and be aware of all the cultural factors that could act as barriers in developing efficient rapport and in eliciting such sensitive information (Rohrabaugh et al. 2016). Fontes (2005) highlighted the most efficient strategy to become familiar with a culture is through “consultation with professionals who come from the culture in question” (p. 71). She stressed the helpfulness of having culture-specific information when working with people from a particular group, since this “alerts us to some of the unique issues that may be important for people from that group and can help us design interventions” (Fontes 2005, p. 11). More research and a better understanding for best practices during child abuse investigations is needed when working with children from various cultures (Barber Rioja and Rosenfeld 2018; Benuto and Garrick 2016).

### Environmental needs

Police stations, where child maltreatment investigations are most often held, often frighten children and may reinforce their feeling that they did something wrong (Cross, et al. 2007). Other possible physical distressors for the child may need to be explored as well. Such as, having an age-appropriate interview room, having the child’s preferred gender to talk with during the interview and ensuring that the person bringing the child to the interview is actually a good support (Allnock and Miller 2013). In Howell et al.’s study (2012), children who felt comfortable showed more ability to engage and better comprehension of what was being asked of them. Similarly, Berson and Meisburger (1998) found that children needed to feel safe and protected in their immediate environment in order for them to disclose. Clearly, investigative interviewers need prior knowledge of the issues that could create challenges for the interviewee during the interview and include appropriate measures to ensure a comfortable and safe space (Davies and Westcott 1999). CYACs are great environments; they were intendedly created as a neutral, child-appropriate, non-intimidating and safe place that can help reduce the stress for the child (Government of Canada 2017; Milam et al. 2017).

### Family dynamics

Several authors tied the importance of understanding the relationship between family dynamics and children’s disclosures during investigative interviews. Lyon, Ahern, Malloy, et al.’s (2010) study revealed that children often expect their caregivers to be unsupportive, rejecting and punitive regarding an abuse disclosure. Research tells us that children of parents who are fearful or who deny the abuse are more likely to demonstrate challenges when interviewed (Hritz et al. 2015): “Children are less likely to disclose and more likely to recant when non-offending parents refuse to believe that abuse occurred or otherwise fail to support the allegation” (Lyon et al. 2012, p. 12). Children who feel isolated from their natural support systems may lead to them recanting a disclosure; interviewers must be aware how family relationship, values, loyalty and familial “secret rules” influence disclosures (Bradley and Wood 1996).

Children with non-offending caregivers who are supportive, on the other hand, demonstrate an ability to disclose much more than those that had non-supportive caregivers (Paine and Hansen 2002). Schaeffer et al.’s (2011) study focused on supporting the victim’s parents/caregivers. Such support may reverse the negative impact the parent’s emotions have on the child, which in turn, then influence on the disclosure. Professionals who provide support to a child’s family may help facilitate disclosures of abuse (Ungar et al. 2009). Supporting the mother after her child initially discloses for example, may be a predictor for memory accuracy. It is suggested how these supportive interventions with the mother could contribute to more credible disclosures for her child (Middleton 2017). Supporting the mother (or other primary caregiver), will also enhance their ability to support their child and increase opportunities for healthier outcomes.

It is important to consider the possible obstacles to disclose abuse in cases where the perpetrator was a parent. Foynes et al. (2009) compared survivors of abuse who were close with the perpetrator with those that were not. Even though there was no significant result with sexual abuse, both emotional abuse and physical abuse demonstrated how victims were significantly more likely to wait one or more year to disclose or never disclose at all. This study may indicate how disclosures of abuse by someone that was close to them could be more traumatic and possibly require more support and empathic responses. Alaggia (2010) shared how young people’s disclosures become more challenging when the child and their parent are both victims of the same perpetrator.

### Uniqueness

Several authors acknowledged that every child has distinctive characteristics, needs and/or limitations and indicate the importance for addressing them within the investigative interview. Disclosures are shaped around the individual’s level of unique set of circumstances, and professionals need to be prepared for disclosures by understanding the child’s obstacles to disclose (Alaggia 2010). An interviewer needs to understand specifics of a child to be able to understand her or his signals indicating when it is safe to push forward with the questioning, when to retreat and what accommodations need to be made (Berson and Meisburger 1998).

Fängström et al. (2017) interesting study explored the use of a computer-assisted tool for shy children during the rapport phase of the interview. These authors discussed how “children who exhibit shy or slow-to-warm-up characteristics react to new people, situations, or contexts with vigilant behaviours and motor quieting; moreover, they are verbally quiet and emotionally reserved. In an interview situation, with an unknown adult, the shy child often behaves in an uncommunicative, anxious, or uncooperative manner” (p. 2). The results of this study showed that the talkativeness increased and the answer latency decreased for the shy children with the computer-assisted interview.

Another example would be the importance for awareness when working with young lesbian, gay or bisexual (LGB) individuals for instance. Unfortunately, lesbian, gay or bisexual, youth can experience many challenges because of how peers or society respond to their sexual orientation (CDC 2021). Studies are starting to demonstrate a growing concern of how sexual minority youth are overrepresented yet may not be adequately served by child welfare services—professionals should be asking children and youth about their identity and learn how their needs could be better understood (Dettlaff et al. 2018).

## Discussion

The literature demonstrates the complex issues encompassing child abuse disclosures. Throughout the literature, it was apparent that the most important source of information about the abuse was from the child. The *findings* highlight the need for interviewers to acknowledge the barriers to disclose in order to understand what may help children and youth tell. Equally apparent, when the interviewer does not address the child’s needs, the ability to disclose becomes challenged. When interviewers enter the forensic interview and are *blind* to the many qualities that make up the child or simply do not have the ability to detect certain impairments, being able to tailor the interview to what the child needs seems unfeasible. Understanding that the child, for instance, is chronologically 12 but cognitively functions as a five year old, is hard of hearing, has difficulty pronouncing the ‘th’ sound, uses a certain slang name to describe the genital area or comes from a culture where talking about private areas are ‘taboo’, may provide some great insight into how the interviewer can prepare for the interview that is specific for what the child may need. Learning about what the child likes or is interested in, would mostly likely help with the initial rapport building; a very crucial aspect of the interview process. Perhaps it would be of benefit to explore the work of Brennan and McElvaney (2020) and McElvaney et al. (2012) regarding children’s therapeutic needs and what they require from adults during disclosures of abuse.

All children deserve a formal disclosure process that maximizes their ability to share information about their abuse. When interviews do not involve a process to enable the witness’s potential, they may unwittingly make things worse—exacerbating the trauma and most likely leading to no disclosure. In most cases, adults have the ability to communicate factors such as feeling scared or unprepared, which may impact their ability to proceed with the interview. But in many cases, the literature revealed, children do not relay such information; they may enter the interview prematurely and/or with uncertainty, thereby increasing the risk of achieving no disclosure. The literature demonstrated that such issues as being scared, in shock, needing information about the investigation procedure, still processing the abuse and/or lacking the vocabulary to describe the abuse, all have the potential to interfere with children disclosing abuse. Children need and deserve time to understand the investigation process and the opportunity to express their challenges in the beginning of this process. If they are unable to communicate their needs, someone must advocate for those needs and enable children to talk comfortably.

### Practice implications

The literature clearly demonstrates a need for the investigation process to slow down prior to the interview and permit time to understand specific circumstances. Literature demonstrated how interviewers need to understand children’s challenges and unique needs so barriers to disclose can be understood, conveyed and addressed within the interview. Forensic interviewing experts have suggested that getting specific information about the child may assist in planning for the interview and in “being sensitive to a child’s specific needs or vulnerabilities” (Rivard and Compo, 2017, p 254). A well-planned investigative interview requires being aware of the many factors and issues unique to the child and of any distinct needs that require support (Davies and Westcott 1999; Newlin et al. 2015; Rohrabaugh et al. 2016). The importance for interviewers to possess such knowledge is not denied; rather, the abundance of responsibility that such a perspective places on the interviewer for successful interview outcomes is questioned. As well, it is questioned how the interviewer might obtain such a sizeable amount of information about children and find the time to do so.

The investigative interview will only be as good as the planning that goes in before the interview is done—prior knowledge becomes paramount (Kebbell et al. 2006; Rohrabaugh et al. 2016). To effectively address all of these expectations means the need to expand thinking from *knowing* there is a need for a tailored interview to understanding *how* one can be achieved. As the literature suggested, when a system of knowledge sharing prior to the interview were included in the investigation process, the interviewer could then prepare a tailored interview specific to each child or youth. Learning about a child’s individual characteristics, competencies and limitations can help interviewers understand how to adapt their approach (Cederborg et al. 2008). The idea of collaboration efforts within MDTs may increase the efficiency of the interview to address case-specific needs, possible barriers and strategies to meet the needs of the child (Newlin et al. 2015). MDTs involve communication collaboration amongst colleagues and is a common practice, allowing for a solid understanding of the multi-faceted needs of children, youth and their families (Anderson-Butcher and Ashton 2004). The use of MDTs in CYACs to assist in gaining such knowledge about the child and in working together to strategize how best to meet his or her needs, would create improvements for children, interviewers and society alike.

#### Improvements for children

Literature indicated the benefits of incorporating MDTs to combine expertise and create a comprehensive understanding of the child’s situation. Many CYACs already have such specialists within their MDTs (such as physicians and mental health clinicians) who specialize in such issues as trauma, and cognitive and physical development (Walsh et al. 2003). Integrating a collaborative effort prior to the interview may heighten the ability for early diagnosis of pertinent mental health concerns requiring immediate intervention. Children who experience such traumatic experiences as abuse often suffer mental health issues that require immediate attention; however, in many cases, they do not get the help they need (Burns et al. 2004). Professionals working together increase the probability of detecting high priority concerns. A fuller understanding of children’s unique circumstances heightens the ability to effectively address them, which then mitigates possibilities for re-traumatization.

#### Improvements for interviewers

The literature undeniably demonstrated the need for highly trained and skilled interviewers, but also to possess specific knowledge about the child, the skills to address specific issues and the ability to create a strategized and tailored interview. This paper suggests enhancing the effectiveness of working in MDTs by creating a team approach to preparing for interviews specific for each child. Having experts and individuals who know the child to provide information on his/her cognitive functioning, communication level, mental and physical health issues, protection needs or cultural aspects can determine the needs within an interview experience. This practice may help the interviewer plan and address these needs and possibly increase opportunity for a child-centred, trauma-informed and culturally safe interview.

The role of a child forensic interviewer is a demanding undertaking; they are tasked with the insufferable duty of uncovering potential acts of violence on children. “Law enforcement professionals… can find cases of child sexual assault [in particular] less desirable to investigate than other crimes” (O’Donohue and Fanetti, 2016, p. 43). Forensic interviewers can face tremendous stress, burnout and the possibility for experiencing vicarious trauma (Perron and Hiltz 2006; Starcher and Stolzenberg 2020). Another advantage for the use of MDTs is that they create a team working together; as a source that provides mutual support for professionals engaged in such emotionally stressful work (Lalayants and Epstein 2005).

#### Improvements for society

Literature revealed how we all bear the responsibility of helping children disclose experiences of abuse, yet the receiver of a disclosure may not know what to do. Other factors that result to withholding information about an abuse disclosure are around avoiding the emotions and confusions that are attached with the thought of reporting. Children may sense this uncertainty in reporting and then interpret it as a silent issue possibly increasing their hesitancy to disclose. It was suggested that more education and awareness were needed to enhance both disclosures and reporting abuse. However, the literature did not indicate what additional education would have such an impact. The literature did demonstrate how the use of MDTs in CYACs could not only improve the investigative process and in helping to understand children’s needs, but can increase awareness around important information. It seems logical that MDTs in CYACs can become resources for society members; a centralized hub one could contact when in question of what to do when a disclosure is received or if one needs to disclose. The identification of one or more community child abuse experts, such as those in CYACs, for consultation may encourage reluctant members of society to call about an incident to work through their doubts and concerns.

## For further thought

The need to integrate intervention prior to the investigative interview is highlighted, to allow awareness and support for children’s immediate needs so potential barriers to disclosures may be decreased. Professionals who work in the area of child abuse are well aware that intervention before the interview is often discouraged (Lipovsky and Hanson 2007). McElvaney’s (2008) study encouraged ethical thinking regarding a child who needs more time to connect or talk with others about the abuse before the interview takes place. Expanding on her thoughts may be to explore the kinds of intervention that could be considered acceptable and would not be identified as possibly tainting evidence. For example, if it was learned that a child used language (appropriate for their cognitive development) during an informal disclosure that resembled sexual abuse (but was not an actual term commonly used)—what could prior intervention look like so both the interviewee and interviewer could prepare for an interview?

Children’s language abilities are interconnected with the development of reasoning, understanding of concepts and emotional maturity. We, therefore, cannot assume that they all possess the ability to describe a horrific, confusing, or complex event (Korkman et al. 2008). North American society encourages abuse education programmes that recommend components that teach children to identify and resist inappropriate touching, reassuring children that it is not their fault and learning the proper names of their genitals (Kenny et al. 2008). However, we cannot speculate that all children would receive this type of training on language regarding abuse. If education occurred before the forensic interview, many may feel this would be the child’s best interest to have him/her able to use and understand language that would allow for a credible disclosure. As most adults have the language to describe what happened to them, it makes sense that children would have this right as well. Yet the concern of suggestibility continues to be a complicated issue regarding children’s disclosures of abuse within the legal system, particularly for interviewers. Klemfuss and Olaguez’s (2020) review was inconclusive for educational/psycho-social affecting children’s suggestibility and encouraged future research in this area. There also appears to be continual disagreement amongst researchers and professionals regarding what and how much information the interviewer should know before conducting the forensic interview (Rohrabaugh et al. 2016; Rivard and Schreiber Compo 2017). Thus, adding to the need for a more comprehensive understanding in this area.

In many CYACs, multidisciplinary intervention begins at the initial report; it includes but is not limited to the pre-interview, which identifies a specific child’s needs (Government of Canada, Department of Justice 2017). Multidisciplinary teamwork can facilitate successful investigations, “while improving the welfare of the children and non-offending caretakers” (MacLeod 2016, p. 43). However, there is no standard of practice for the composition of the team or approaches to collaboration; therefore, the members of an MDT and their functions vary across communities. The same is true for the CYAC practice in designating a child victim service worker or advocate as on-going support for a child and his/her family, and in most cases, as the first point of contact for them. When children first participate in the investigative process, professionals must have specialized training in the dynamics of child abuse and trauma; otherwise, misinterpretation of their state may create risk for further traumatization, thereby reducing children’s ability to communicate (National Child Traumatic Stress Network 2008). Such professionals who work with children immediately following a report of abuse are interacting with them at a critical moment. Including discussions with the child’s guardians or other professionals who work and know the child is beneficial as well, to gain insight into their developmental level or concerning issues (Rohrabaugh et al. 2016). It may have been beneficial for this paper to explore how victim service workers/advocates and MDTs in CYACs who specialize in child abuse and trauma, work together prior to the investigative interview. Their approaches may help us understand how and if their interventions have an effect or influence children’s disclosures during the interview. Lippert et al. (2009) study demonstrated that the rates of children’s disclosures were similar between communities that had a CYAC and those that did not. However, these authors also encouraged further research to examine the influence of “setting characteristics, interviewers’ background (forensic interviewer vs. CPS vs. police), interview methods, the diversity of cases, and methodological differences on disclosure rates” (p. 110–111).

Another area that would benefit from further exploration is disclosure issues among Indigenous children and youth. Research regarding issues that impact disclosures specifically with Indigenous children and youth was not found. Due to colonization (past and present), many Indigenous children endure both developmental and intergenerational components, adding to the complexities of trauma (Ministry of Children and Family Development 2017). Literature most often addressed barriers to disclose as inclusive of all nationalities; perhaps these generalizations add to the factors that create challenges and issues to disclose among Indigenous children? Some aspects specific to Canadian Indigenous culture may influence whether children withhold a disclosure, especially when factors such as intergenerational trauma are considered (Collin-Vézina et al. 2009). It would have been beneficial to have Indigenous-specific research included in this review to understand issues that impact disclosures of abuse pertaining to Canadian Indigenous children. It would also be interesting to explore research regarding the use of MDTs and CYACs with different cultural views to better understand culturally safe practices when responding to cases of child maltreatment.

## Conclusion

The literature clearly demonstrated the complex issue regarding child abuse disclosures. There are numerous factors to consider when responding to cases of child maltreatment and the issues that have an impact on children’s ability to disclose during the investigative interview. Rapport with the interviewer, the child’s need to feel empowered and have access to information, communication barriers, developmental abilities or limitations, mental health issues, environment needs, family dynamics, cultural issues and just needing an understanding of the child’s specific circumstances or other issues that can interfere with the ability to disclose. Literature clearly demonstrated how the factors that influence children’s disclosures are loaded conversations themselves. Each factor requires further exploration to fully appreciate how many issues there are in each and how combinations of factors create a multi-faceted effect impacting children during their disclosures of abuse.

It is evident that children need help to talk about their abuse, and this makes it a societal issue; this is where encouragement to talk about abuse begins. The disadvantages of a nondisclosure can be enormous for the victim and their family, and the long-term consequences are very costly for society. The review suggested that the investigative process slow down prior to the interview, to allow time to understand the child’s circumstances and tailor the interview to suit their needs. Perhaps a good starting place is to stop viewing issues as obstacles, rather as indicators and opportunities to understand what each specific child needs during disclosures of abuse.

A thorough gathering of background information before the interview is suggested, by utilizing the expertise and wisdom of MDTs and other community partners as needed. The literature revealed that MDTs and CYACs were ideal resource systems to incorporate the combination of expertise working and communicating together to best understand and meet the needs of the child. MDTs and CYACs could also be identified as a central resource for societal members needing to consult about child abuse cases that may help them encourage young victims to formally disclose abuse.

We still have a lot to learn about most effective, trauma-informed and culturally safe approaches when responding to cases of child maltreatment that fully meet the needs of the child. Perhaps another good starting point is to start asking ourselves if we have enough information about the child that permits us to say we are child-centred, trauma-informed and culturally safe? The saying (traditionally an African proverb, now adopted by many North American Indigenous communities), i*t takes a whole village to raise a child,* relates to what this review demonstrates regarding the need for MDTs, CYACs, welfare/legal systems and society working together, as it takes a community to help children to disclose their abuse (Pence 1999).
